# Level of arterial ligation in sigmoid colon and rectal cancer surgery

**DOI:** 10.1186/s12957-016-0819-3

**Published:** 2016-04-01

**Authors:** Koji Yasuda, Kazushige Kawai, Soichiro Ishihara, Koji Murono, Kensuke Otani, Takeshi Nishikawa, Toshiaki Tanaka, Tomomichi Kiyomatsu, Keisuke Hata, Hiroaki Nozawa, Hironori Yamaguchi, Shigeo Aoki, Hideyuki Mishima, Tsunehiko Maruyama, Akihiro Sako, Toshiaki Watanabe

**Affiliations:** Department of Surgical Oncology, Faculty of Medicine, the University of Tokyo Hospital, 7-3-1 Hongo, Bunkyo-ku, Tokyo, 113-0033 Japan; Department of Surgery, Hitachi General Hospital, 2-1-1 Jonan,, Hitachi, Ibaraki Pref Japan

**Keywords:** Rectal cancer, Sigmoid colon cancer, High tie, Low tie, Prognosis

## Abstract

**Background:**

Curative resection of sigmoid colon and rectal cancer includes “high tie” of the inferior mesenteric artery (IMA). However, IMA ligation compromises blood flow to the anastomosis, which may increase the leakage rate, and it is unclear whether this confers a survival advantage. Accordingly, the IMA may be ligated at a point just below the origin of the left colic artery (LCA) “low tie” combined with lymph node dissection (LND) around the origin of the IMA (low tie with LND). However, no study has investigated the detailed prognostic results between “high tie” and “low tie with LND.” The aim of this study was to assess the utility of “low tie with LND” on survival in patients with sigmoid colon or rectal cancer.

**Methods:**

A total of 189 sigmoid colon or rectal cancer patients who underwent curative operation from 1997 to 2007 were enrolled in this study. The patient’s medical records were reviewed to obtain clinicopathological information. Overall survival (OS) and relapse-free survival (RFS) rates were calculated using the Kaplan-Meier method, with differences assessed using log-rank test.

**Results:**

Forty-two and 147 patients were ligated at the origin of the IMA (high tie) and just below the origin of the LCA combined with LND around the origin of the IMA (low tie with LND), respectively. No significant differences were observed in the complication rate and OS and RFS rates in the two groups. Further, no significant difference was observed in the OS and RFS rates in the lymph node-positive cases in the two groups.

**Conclusions:**

“Low tie with LND” is anatomically less invasive and is not inferior to “high tie” with prognostic point of view.

## Background

The problem of whether to tie off the inferior mesenteric artery (IMA) at its origin (high tie) or just below the origin of the left colic artery (LCA: low tie) in radical surgery for sigmoid colon and rectal cancer has long been debated, but thus far, no clear consensus has been achieved, and the level of arterial ligation still varies among institutions and patients [1]. In oncological terms, high tie has been found to enable full lymph node dissection (LND) and to make a greater contribution to accurate staging [2–4]. When creating an anastomosis between the proximal colon and the remaining rectum or anus in anus-preserving surgery, the mesocolon must be extended to minimize the tension placed on the anastomosis, and division of the IMA at its origin has been reported to be effective in this respect [5]. The degree to which it increases anastomotic leakage by reducing blood flow to the resected margin of the intestine has not been addressed in any previous study. After a high tie is performed, perfusion to the proximal colon is supplied solely by the superior mesenteric artery, and decreased anastomotic perfusion is thus a matter of concern [6, 7]. Other studies have also found that high tie may increase the risk of autonomic nerve damage around the origin of the IMA [8].

According to data from the Japanese Society for Cancer of the Colon and Rectum, the rate of positive lymph node (LN) metastases around the origin of the IMA is 3.6 % in pT3/T4 sigmoid colon cancer and 5.1 % in rectal cancer, numbers that are not negligible [9]. In Europe and the USA, surgical procedures in which the vessel is resected at sites other than the area of LND are not generally used. In Japan, however, surgical procedures are widely used in which lymph nodes are dissected along the vessel, and the ligation and division of a blood vessel distal to the area is commonly performed. With respect to LND around the origin of the IMA and the site of division, a widely utilized surgical procedure is to carry out LND around the origin of the IMA on the central side and to preserve the LCA with the aim of preserving anastomotic perfusion.

Preservation of the LCA and LND around the origin of the IMA is widely performed in Japan [10]. This method is used in consideration of the significance of the lymph node dissection around the origin of the IMA by means of ligation of the origin of the IMA, as well as the significance of preserving anastomotic perfusion by preservation of the left colic artery. However, a comparative analysis of the outcomes of patients treated with high tie and those treated with low tie combined with the lymph node dissection around the origin of the IMA (low tie with LND) has yet to be performed out. In this study, we investigated whether the outcomes of patients who underwent a low tie in addition to dissection of the lymph nodes around the origin of the IMA were inferior to the outcomes of those who underwent a high tie.

## Methods

**Fig. 1:**
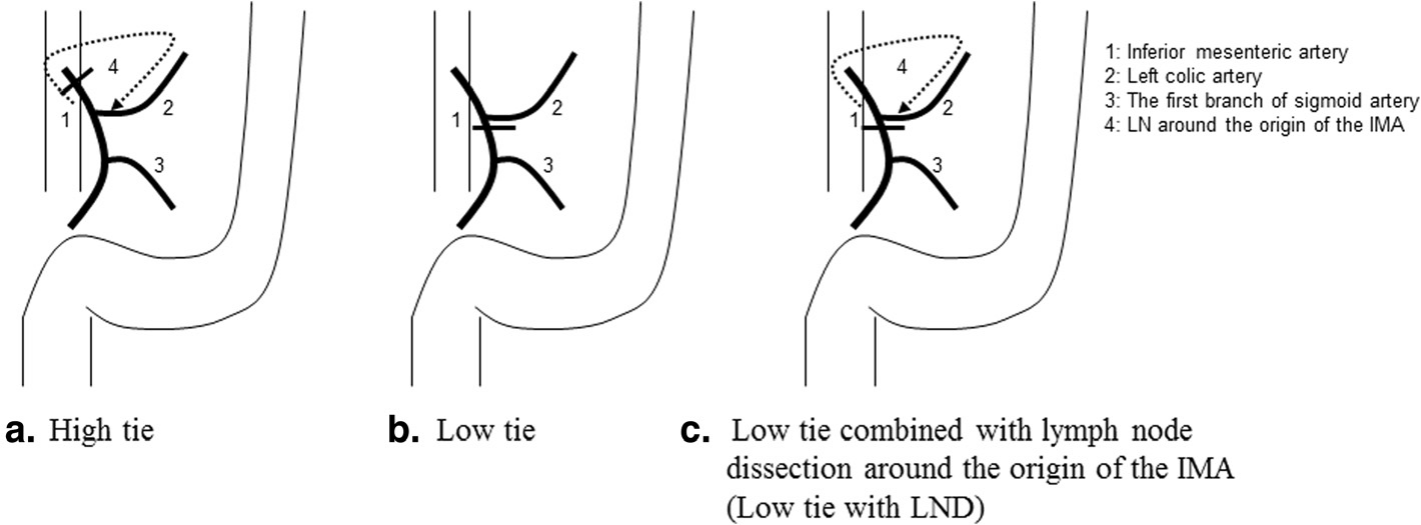
Surgical technic schema of the two groups (**a**: High tie, **b**: Low tie, **c**: Low tie combined with lymph node dissection around the origin of the IMA)

The study subjects were 189 patients who underwent surgery for sigmoid colon cancer or rectal cancer between January 1997 and March 2007. They were divided into the following two groups: patients in the high tie group (*n* = 42) underwent ligation at the root of the IMA (high tie), whereas those in the low tie with LND group (*n* = 147) underwent ligation just below the origin of the LCA combined with LND around the origin of the IMA (Fig. 1). We excluded stage 0 and IV and non-curative patients from this study. The selection of the level of IMA ligation was decided by an operator. All cases were operated by an open method.

We performed a retrospective analysis of these two groups considering clinicopathological factors, clinical data, complications, recurrence, and survival. The clinicopathological factors included location, histological depth, lymph node metastasis, lymphatic duct invasion, venous invasion, and stage. The clinical data included operating time, blood loss, duration of postoperative hospitalization, number of patients with lymph node metastasis, and number of lymph node dissection. The information on complications considered the incidence and type, and the data on recurrence included the recurrence rate and types of organs involved. Multivariate analyses of the factors that might influence the overall and relapse-free survival were performed. We also evaluated the 5-year overall survival (OS) and relapse-free survival (RFS) rates of all patients and patients with positive lymph node metastasis.

Postoperative follow-up examinations were serum carcinoembryonic antigen (CEA) and serum carbohydrate antigen 19-9 (CA19-9) measurements and computed tomography every 6 months and lower gastrointestinal endoscopy every year. All patients underwent postoperative follow-up for 5 years. Histopathological diagnosis of surgical specimens was performed by a pathologist, and pathological assessment was performed on the basis of the UICC TNM Classification (7th edition). Stage 0 and stage IV patients were excluded from the study. All study procedures were performed in accordance with the Declaration of Helsinki. The Kaplan-Meier method was used to analyze OS rates of all patients and RFS rates of patients with positive lymph node metastasis, and significant differences were analyzed by using a log-rank test. *χ*^2^ test and Mann-Whitney *U* test were used for the statistical analyses shown in the other tables. Statistical analysis was performed with GraphPad Prism 5 software (GraphPad Software Inc. San Diego, CA, USA) and JMP 11.2 software (SAS Institute Inc. Cary, NC, USA).

## Results

### Clinicopathological factors

Table 1 shows various clinicopathological factors for both two groups. No statistically significant differences were observed in sex, age, tumor location, depth, N factor, lymphatic invasion, venous invasion, or stage between the two groups (Table 1).

**Table 1:** Clinicopathological background of patients

	Group A (*n* = 42)	Group B (*n* = 147)	*P* value
M/F	26/16	92/55	0.936
Age (year) ± SD	64.5 ± 9.6^a^	68 ± 9.1^a^	0.695
Tumor location			
Sigmoid colon	17 (40.5 %)	56 (38.1 %)	0.086
Rectosigmoid	8 (19 %)	30 (20.4 %)	
Upper rectum	10 (23.8 %)	36 (24.5 %)	
Lower rectum	7 (16.7 %)	25 (17 %)	
Depth			
T1	1 (2.4 %)	13 (8.8 %)	0.079
T2	5 (11.9 %)	35 (23.8 %)	
T3	15 (35.7 %)	63 (42.9 %)	
T4	21 (50 %)	36 (24.5 %)	
N			
0	22 (52.4 %)	81 (55.1 %)	0.400
1	17 (40.5 %)	50 (34 %)	
2	3 (7.1 %)	16 (10.9 %)	
Lymphatic invasion			
Absent	7 (16.7 %)	19 (12.9 %)	0.535
Present	35 (83.3 %)	128 (87.1 %)	
Venous invasion			
Absent	25 (59.5 %)	97 (66 %)	0.440
Present	17 (40.5 %)	50 (34 %)	
Stage			
I	2 (4.8 %)	38 (25.9 %)	0.100
II	21 (50 %)	44 (29.9 %)	
III	19 (45.2 %)	65 (44.2 %)	

Group A: high tie. Group B: low tie combined with lymph node dissection around the origin of the inferior mesenteric artery (low tie with LND)

*n*: number, *M*: male, *F*: female, *N*: regional lymph node

^a^Average ± standard deviation

### Clinical data

The operative time was 204 min in the high tie group and 190 min in the low tie with LND group, and the difference in operative time was not statistically significant between the two groups. Additionally, there were no statistically significant differences in the amount of blood loss and the number of days between the two groups. The number of lymph node metastasis-positive cases was 20 (47.7 %) in the high tie group and 67 (45.6 %) in the low tie with LND group, whereas the number of LND (per person) was 15.5 in the high tie group and 13 in the low tie with LND group, with no statistically significant differences in the number of lymph node metastasis-positive between the two groups. In addition, the number of cases positive for LN at the root of the IMA metastasis was 2 (4.8 %) in the high tie group and 3 (2.0 %) in the low tie with LND group, with no statistically significant difference in this number between the two groups (Table 2).

**Table 2:** Clinical data of patients

	Group A (*n* = 42)	Group B (*n* = 147)	*P* value
Operation time (min)	204	190	0.425
Blood loss (g)	160	120	0.158
Number of patients with a metastatic LN^a^	20 (47.7 %)	67 (45.6 %)	0.815
Number of harvested LN/patient	15.5	13	0.184
Number of patients with metastatic LN root of the IMA	2 (4.8 %)	3 (2.0 %)	0.333
Postoperative hospitalized days	20.03	18.68	0.163

*LN*: lymph node, *IMA*: inferior mesenteric artery

^a^Median

### Postoperative complications

Complications developed in 8 patients (19.0 %) in the high tie group and 25 patients (17.0 %) in the low tie with LND group, and the difference in the complication rate between the two groups was not statistically significant. The most common complication was ileus in the high tie group (4 cases) and surgical site infection (SSI) in the low tie with LND group (13 cases). No statistically significant differences were observed in the number of complications between the two groups (Table 3).

**Table 3:** The characteristics of the complication

	Group A (*n* = 42)	Group B (*n* = 147)	*P* value
Complication	8 (19.0 %)	25 (17.0 %)	0.759
SSI	2	13	0.388
Ileus	4	8	0.339
Anastomotic leakage	2	3	0.333
Urinary infection	0	1	0.592
Urinary dysfunction	1	0	0.592

*SSI*: surgical site infection

### The assessment of the rate and organ of recurrence

There were 10 (23.8 %) cases of recurrence in the high tie group and 30 (20.4 %) cases of recurrence in the low tie with LND group, with no statistically significant difference in this number between the two groups. Lymph node recurrence occurred in 2 (4.8 %) cases in the high tie group and 5 cases (3.4 %) in the low tie with LND group, and the difference in this recurrence rate was not statistically significant between the two groups. The most common organs of recurrence were the liver and lungs in the high tie group with 4 cases each (9.5 %) and the lungs in the low tie with LND group with 9 cases (6.1 %). No statistically significant differences were observed for any of the recurrence sites between the two groups (Table 4).

**Table 4:** Recurrent organ

	Group A (*n* = 42)	Group B (*n* = 147)	*P* value
Recurrence	10 (23.8 %)	30 (20.4 %)	0.634
Liver	4 (9.5 %)	8 (5.4 %)	
Lung	4 (9.5 %)	9 (6.1 %)	
LN	2 (4.8 %)	5 (3.4 %)	
Para-aortic LN	2 (4.8 %)	3 (2.0 %)	
Internal iliac artery LN	0	2 (1.4 %)	
Local	3 (7.1 %)	8 (5.4 %)	
Bone	0	5 (3.4 %)	
Peritoneum	1 (2.4 %)	3 (2.0 %)	

*LN* lymph node

### Prognosis

**Fig. 2:**
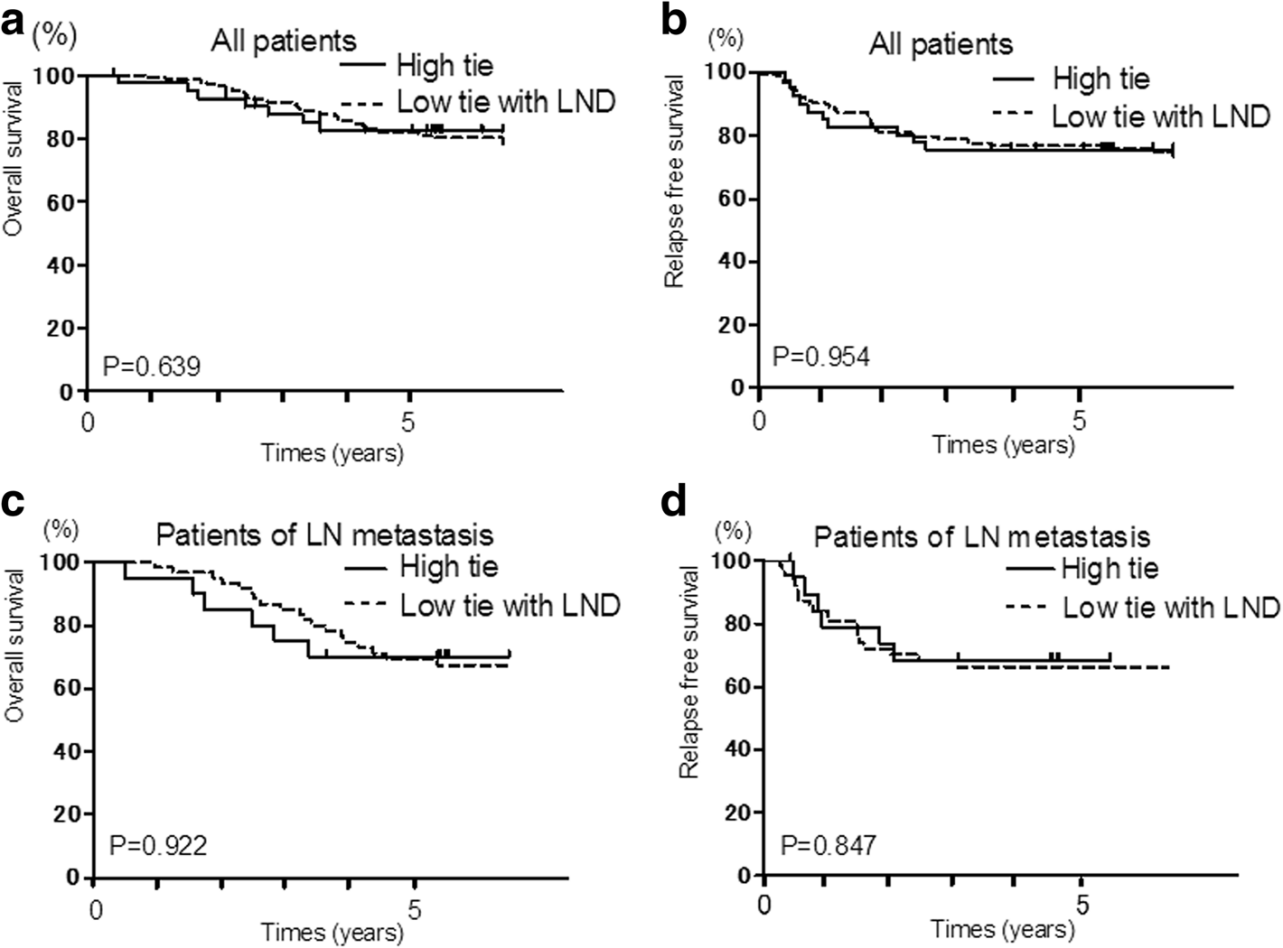
Overall survival (OS) and relapse-free survival (RFS) rates of colon cancer patients by Kaplan-Meier analysis. **a** Kaplan-Meier survival curves for OS in all patients between both groups do not have a significant difference. **b** Kaplan-Meier survival curves for RFS in all patients between both groups do not have a significant difference. **c** Kaplan-Meier survival curves for OS in lymph node-positive patients between both groups do not have a significant difference. **d** Kaplan-Meier survival curves for RFS in lymph node-positive patients between both groups do not have a significant difference. (*P* < 0.05)

In both the high tie group and low tie with LND group, the 5-year survival rate was 82.4 and 80.3 %, respectively, whereas the recurrence-free survival rate was 75.6 and 76.2 %, respectively, with no statistically significant differences in these rates between the two groups (Fig. 2a, b). Additionally, for cases of lymph node metastasis in the two groups, the 5-year survival rate was 70.0 and 67.4 %, respectively, whereas the recurrence-free survival rate was 68.4 and 66.3 %, respectively, and the differences in these rates were not statistically significant between the two groups (Fig. 2c, d).

### Multivariate analysis of prognostic factors

Multivariate analyses for overall survival and relapse-free survival are shown in Tables 5 and 6. Age was associated with overall survival, and T and N stages were associated with relapse-free survival; however, the level of IMA ligation was not significantly associated with overall and relapse-free survival (Tables 5 and 6).

**Table 5:** Multivariate analysis of overall survival

			Multivariate	
		HR	95 % CI	*P* value
Sex				0.6394
	Female	1		
	Male	1.2	0.56–2.74	
Age		1.06	1.02–1.11	0.0071^†^
Tumor location				0.4685
	Sigmoid	1		
	Rectosigmoid	0.99	0.33–2.71	
	Upper rectum	1.26	0.49–3.23	
	Lower rectum	2.27	0.77–6.56	
T stage				0.0681
	T1	1		
	T2	0.43	0.05–9.24	
	T3	1.87	0.31–36.19	
	T4	1.69	0.27–33.31	
N stage				0.5535
	N0	1		
	N1	1.52	0.68–3.44	
	N2	1.48	0.47–4.24	
Lymphatic invasion				0.6929
	Absent	1		
	Present	0.76	0.22–3.54	
Venous invasion				0.3709
	Absent	1		
	Present	1.41	0.56–2.99	
Operation				0.3882
	High tie	1		
	Low tie with LND	1.56	0.59–4.96	

*HR*: Hazard ratio, *CI*: Confidence interval

^†^Significant difference

**Table 6:** Multivariate analysis of relapse-free survival

			Univariate	
		HR	95 % CI	*P* value
Sex				0.6482
	Female	1		
	Male	0.84	0.41–1.79	
Age		1.04	1.00–1.08	0.0265^†^
Tumor location				0.1287
	Sigmoid	1		
	Rectosigmoid	1.73	0.59–4.91	
	Upper rectum	3	1.21–7.90	
	Lower rectum	1.92	0.64–5.66	
T stage				0.0101^†^
	T1	1		
	T2	1.16 × 10^9^	0.19	
	T3	7.36 × 10^9^	0.79	
	T4	5.67 × 10^9^	1.22	
N stage				0.0223^†^
	N0	1		
	N1	1.56	0.71–3.52	
	N2	4.09	1.53–10.69	
Lymphatic invasion				0.905
	Absent	1		
	Present	0.92	0.26–4.32	
Venous invasion				0.1857
	Absent	1		
	Present	1.59	0.80–3.20	
Operation				0.9103
	High tie	1		
	Low tie with LND	0.95	0.24–2.30	

*HR*: Hazard ratio, *CI*: Confidence interval

^†^Significant difference

## Discussion

The ligation level of the IMA in radical operations treating sigmoid colon cancer or rectal cancer, whether it should be ligated at the root of the IMA (high tie) or just below the origin of the LCA (low tie), has been discussed domestically and internationally. However, an unambiguous consensus remains to be achieved [11]. In Japan, the concept of LND is widely accepted, and D3 LND is a standard treatment whereas D2 dissection is permitted only in patients in whom the tumor invasion depth was found to be restricted to the muscular layer during preoperative diagnosis and who did not have lymph node metastases [12]. Consequently, lymph node involvement along the root of the IMA is dissected in many patients and the concept of high tie has been widely adopted as the gold standard. In contrast, there are many institutions where the operative procedure selected is to preserve the LCA to maintain anastomotic blood flow, to prevent an anastomotic leakage after colectomy, which is an insurmountable issue even as surgical techniques of colon cancer have advanced.

It has been reported that the incidence of anastomotic leakage after surgery for rectal cancer is 5–26 % [13–15]. Ensuring the anastomosis is tension-free and maintaining blood flow is believed to be important to reduce this incidence [16, 17]. And the level of IMA ligation can determine the “reach” of proximal colon to be anastomosed [18]. High tie, enabling anastomotic tension to be released, is superior to low tie ligation. Low ligation can prolong the reach by increased blood supply; however, in turn, it can hamper the reach by the tension of the mesentery due to the remnant LCA. In addition, it has been reported that the rate of positive lymph nodes at the root of the IMA in patients with rectal cancer is 4.9 % (0.3–11.1 %) [11]. Furthermore, a study revealed that high tie dissecting lymph node involvement around the root of the IMA is acceptable from the viewpoint of rectal cancer prognosis based on lymph node excision [19]. However, it has recently been reported that there is no clear significant difference in prognosis between the high tie and low tie procedures [5, 20, 21]. A high tie is more likely to damage the nerve plexus around the IMA root, resulting in autonomic nervous system disorders such as urinary dysfunction [22]. Furthermore, it has been reported that blood flow from the IMA is impaired by a high tie and blood flow to the oral side of the anastomosis must rely on the middle colic artery, leading to reduced blood flow [6, 7]. Meanwhile, another study reported that a low tie promotes anastomotic blood flow and oxygenation and reduces the local recurrence associated with suture failure [23]. One additional advantage of this operative procedure is that since there is blood flow of the LCA, the surgery of the residual colon is enabled, and in cases where secondary carcinogenesis occurs in the preserved ascending colon or transverse colon in patients who had received surgery for sigmoid colon cancer or rectal cancer, the left transverse colon can be preserved because the LCA remains. We may have a potential to preserve a left side transverse colon because LCA is preserved. Based on these previous studies, there is a widely accepted operative procedure, low tie with LND around the origin of the IMA, in Japan. This procedure involves IMA root dissection focusing on the importance of D3 LND, which is combined with the preservation of the LCA enabling the important preservation of anastomotic blood flow. This procedure is different from the standard low tie. The incidence of complications other than anastomotic leakage and the length of hospital stay also did not differ significantly in both groups. The hospital stay seems to be prolonged. This might be due to the unique situation in Japan that majority of the medical cost is covered by public health insurance. There are extremely few reports comparing high tie and this procedure from the viewpoints of complication rate and prognosis.

In this study, encompassing 189 patients with sigmoid colon cancer or rectal cancer who underwent radical operation, the complication rate, recurrence rate, and prognosis were compared between the high tie group and the low tie with LND group. As a result, the incidence of anastomotic leakage was not significantly greater in the low tie with LND group than in the high tie group. These results supported previous reports showing that the difference between IMA ligation levels was barely involved in the incidence of anastomotic leakage [24]. No significant difference was found in the incidence of urinary dysfunction between the two groups. Additionally, no significant difference was observed for the 5-year survival rate and RFS rate in all patients. For node-positive patients, the 5-year survival rate and RFS rate also showed no significant difference between both groups. These results demonstrated the reliability of LND around the origin of the IMA, enabling ligation of the IMA at the more distal side than the origin of the LCA, commonly performed in Japan. These results also were similar to previous reports, finding no significant difference in the prognosis of the high tie and low tie groups [25, 26].

The most important limitations of this study are the small size of the study population and retrospective nature of the study design. There is a possibility that high tie of the IMA improved the prognosis of the patients with more advanced disease, and therefore, the prognosis of both patient groups was equivalent. However, the background of the patients did not differ significantly, although there was a tendency of more advanced T stage in the high tie group, and multivariate analysis revealed no significant impact of the level of IMA ligation on prognosis. In addition, circumferential resection margin (CRM) and completeness of total mesorectal excision (TME) were not evaluated in this study, and this is also a limitation of this study. It might be possible that the high tie group included much advanced disease. However, the significant difference was not found in the cancer stage in both groups in this study (Table 1). We think that a large scale of randomized controlled study is originally necessary.

## Conclusions

Based on these results obtained by performing radical operation for sigmoid colon cancer or rectal cancer, the low tie with LND around the origin of the IMA procedure is believed to be thoroughly acceptable, considering prognosis. This examination is a retrospective study, and it is expected that a large-scale randomized controlled trial will be performed to determine the position of ligation of the IMA during radical operation for sigmoid colon cancer or rectal cancer.

## Abbreviations

CA19-9: carbohydrate antigen 19-9
CEA: carcinoembryonic antigen
CRM: circumferential resection margin
IMA: inferior mesenteric artery
LCA: left colic artery
LN: lymph node
LND: lymph node dissection
OS: overall survival
RFS: relapse-free survival
SSI: surgical site infection
TME: total mesorectal excision
